# Autosomal Recessive Congenital Sensorineural Hearing Loss due to a Novel Compound Heterozygous PTPRQ Mutation in a Chinese Family

**DOI:** 10.1155/2018/9425725

**Published:** 2018-04-19

**Authors:** Xia Wu, Shan Wang, Sen Chen, Ying-ying Wen, Bo Liu, Wen Xie, Dan Li, Lin Liu, Xiang Huang, Yu Sun, Wei-jia Kong

**Affiliations:** ^1^Department of Otorhinolaryngology, Union Hospital, Tongji Medical College, Huazhong University of Science and Technology, Wuhan 430022, China; ^2^Department of Obstetrics and Gynecology, Union Hospital, Tongji Medical College, Huazhong University of Science and Technology, Wuhan 430022, China; ^3^Institute of Otorhinolaryngology, Union Hospital, Tongji Medical College, Huazhong University of Science and Technology, Wuhan 430022, China; ^4^Hubei Medical Center of Otorhinolaryngology-Head and Neck, Wuhan 430022, China

## Abstract

PTPRQ gene, encoding protein tyrosine phosphatase receptor Q, is essential for the normal maturation and function of hair bundle in the cochlea. Its mutations can cause the defects of stereocilia in hair cell, which lead to nonsyndromic sensorineural hearing loss. Using next-generation sequencing and Sanger sequencing method, we identified a novel compound heterozygous missense mutation, c.4472C>T p.T1491M (maternal allele) and c.1973T>C p.V658A (paternal allele), in PTPRQ gene. The two mutations are the first reported to be the cause of recessively inherited sensorineural hearing loss. Hearing loss levels and progression involved by PTPRQ mutations among the existing cases seem to be varied, and the relationship between genotypes and phenotypes is unclear. Our data here further prove the important role of PTPRQ in auditory function and provide more information for the further mechanism research of PTPRQ-related hearing loss.

## 1. Introduction

Hearing loss is one of the most common sensory disabilities in humans. According to the latest data of WHO, there are 360 million people—over 5% of the world's population—suffering from hearing loss, with 32 million are children (http://www.who.int/mediacentre/factsheets/fs300/en/). Genetic factors are the major cause of congenital sensorineural hearing loss (SNHL). Approximately 80% of nonsyndromic genetic hearing loss is autosomal recessive inheritance [1]. Currently, 64 genes for autosomal recessive nonsyndromic SNHL have been mapped (http://hereditaryhearingloss.org).

PTPRQ gene, encoding protein tyrosine phosphatase receptor Q, is one of the latest identified causes accounting for nonsyndromic SNHL. It is assigned DFNB84 locus on chromosome 12q21.31 and comprised of 58 exons [2]. The PTPRQ protein, localized in the basal region of the stereocilia membrane, is one of the membrane proteins which composed of 2299 amino acids. It has been reported that PTPRQ may have key roles in hair cells: establishing the membrane at the base of the stereocilia, regulating actin dynamics, and tethering the stereocilia membrane to the cytoskeleton with Myosin VI [3–5]. It is known to be required for the development of hair bundles, regulation of normal maturations, and formation of shaft connectors [6].

To date, there are eight families with inherited recessive mutations of PTPRQ which have been published [2, 7–10]. Identifications of PTPRQ mutations could be helpful to establish a better understanding of the relationship between PTPRQ and SNHL. Here, we present a Chinese family with congenital SNHL caused by a novel compound heterozygous PTPRQ mutation.

## 2. Materials and Methods

### 2.1. Family Description

**Figure 1:**
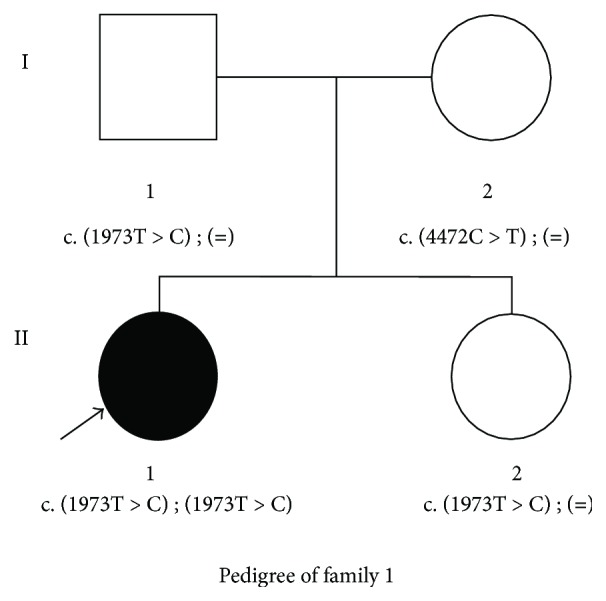
Pedigree of the affected Family 1 with congenital SNHL. Sanger sequencing analysis showed that patient II1 had the compound heterozygous mutation (c.[1973T>C]; c.[4472C>T]), and the parents and sister had the heterozygous mutation. The patient is denoted in black. [=] means wild type.

This Chinese family, named Family 1, is a two-generation family associated with autosomal recessive nonsyndromic SNHL (Figure 1). The affected member II1, a 4-year and 2-month-old child, was diagnosed with congenital SNHL. The other individuals (I1, I2, and II2) had no history of hearing impairment. The child who was born in Hubei Province failed the newborn hearing screening and was diagnosed as congenital sensorineural hearing loss.

### 2.2. Audiological Examination

Visual reinforcement audiometry (VRA) was performed after the patient underwent otoscopic examination in our department. Degree of hearing loss was assessed by using pure tones. The stimuli were produced in the frequencies of 0.25, 0.5, 1, 2, 4, and 6 kHz. By using the stimulus-reply-visual reinforcement conditioning, the minimum response level was obtained in the lowest intensity which the child responded.

### 2.3. DNA Preparation

Peripheral venous blood samples from all the family members were obtained for genetic analysis. Genomic DNA was extracted from the blood samples using QIAamp DSP DNA Blood Mini Kit (61104, Qiagen Inc., Germany) according to kit's protocol.

### 2.4. Next-Generation Sequencing + Sanger Sequencing

The target deafness-related gene capture and next-generation sequencing + Sanger sequencing were performed by MyGenostics Inc. (Beijing, China). First, the genomic DNA was fragmented to special size about 350–400 base pair for library construction. End-repair and Illumina adapter ligation were taken according to the Illumina protocols. After PCR amplification, target DNA fragments were captured with biotinylated single-strand DNA capture probe (MyGenostics, MD, USA) by hybridization. The target gene fragments were enriched, and then high-throughput sequencing was performed using Illumina HiSeq2000 Analyzer for automated cycles per read. Primary data were generated using Trim-Galore software (version 0.4.3). Reads were matched to NCBI37/hg19 using BWA program. Previously identified SNPs were annotated using CCDS, human genome database (HG19), and dbSNP (v138). SIFT and POLYPHEN2 were utilized to predict the function of SNP-affected protein.

### 2.5. Structural-Based Model Building and Analysis

The molecular homology modeling of the human wild type and mutations was built up by SWISS-MODEL (http://www.swissmodel.expasy.org/). The complete protein sequence of human PTPRQ is available in the NCBI GenBank (NP_001138498.1). Data were showed by JavaScript Protein Viewer.

## 3. Results

### 3.1. Mutation Detection and Analysis

**Figure 2:**
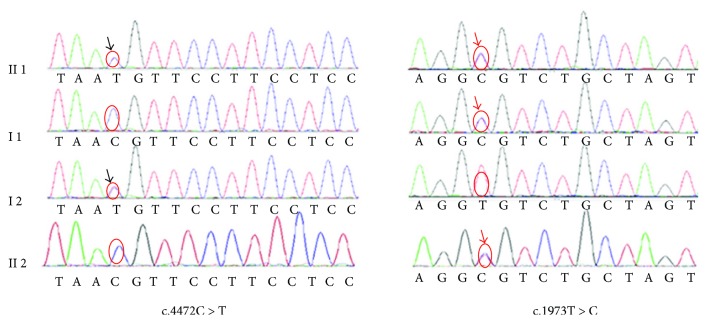
Electropherogram analysis of PTPRQ in Family 1. Electropherogram analysis showed that compound heterozygous mutations (c.4472C>T, black arrows and c.1973T>C, red arrows) cosegregate with the phenotype.

Mutations in mitochondria and miRNA regions were excluded. After aligning to the human reference genome (GRCh37/hg19), these mutations corresponded to c.4472C>T and c.1973T>C, occurring in exon26 and exon13 of PTPRQ. The c.4472C>T leads to a single substitution within the fibronectin type III (FNIII) domain, from threonine to methionine (p.T1491M), and the other, c.1973T>C, leads to a single substitution (valine to alanine; p.V658A) within the FNIII domain. SIFT (http://sift.jcvi.org/) and POLYPHEN2 (http://genetics.bwh.harvard.edu/pph2/) were used to analyze the amino acid substitutions of p.T1491M and p.V658A. Both programs predicted these two mutations to be deleterious, which means they probably damage and affect protein functions. The sequencing results showed that the two parents were heterozygous carriers of c.4472C>T (maternal allele) and c.1973T>C (paternal allele), which demonstrated the compound heterozygous cosegregating mutation with the phenotype in II1 (Figure 2). The frequency of c.4472C>T mutation is 0.0016 in the East Asian population of EXAC database as well as 0.0002 in 1000 Genome Project. The c.1973T>C mutation rate accounts for 0.0222 in 1000 Genome Project. The frequency of c.1973T>C mutation was not found in the East Asian population of EXAC database.

### 3.2. Structure Modeling

**Figure 3:**
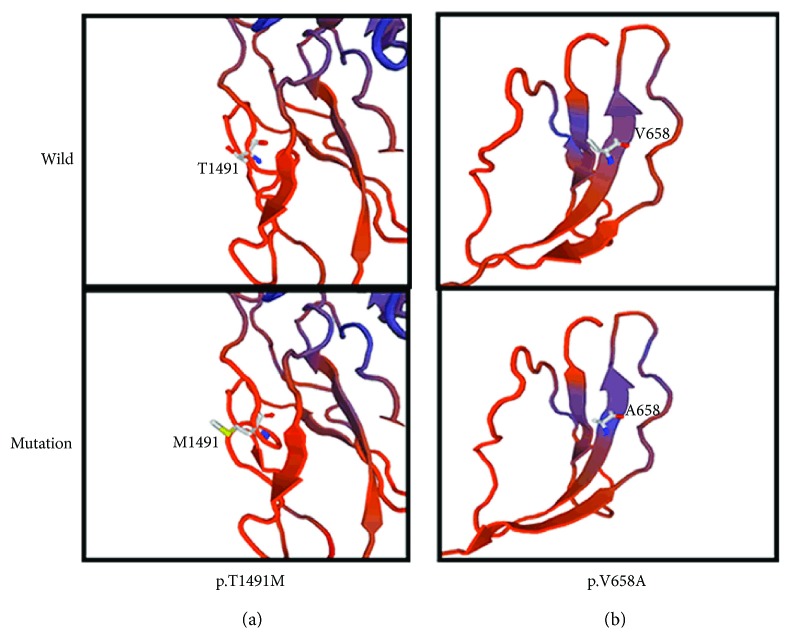
Protein molecular models of wild types and PTPRQ mutations. (a) The mutation protein M1491 has a different side chain to the wild-type protein T1491. (b) The wild-type protein has a longer side chain than the mutation protein A658.

Protein tertiary structures were modelling with SWISS-MODEL (http://www.swissmodel.expasy.or-g/), which predict the sequence homology. The p.T1491M protein model, covering the target sequence (residues 1163–1564), was constructed based on the receptor-type protein tyrosine phosphatase S (PDB ID: 4pbx.1.A). Sequence identity between the target and template was 25.78%. As shown in Figure 3(a), the mutation probably affected the amino acid side chain through the substitution of threonine acid to methionine. The p.V658A protein model, covering the target sequence (residues 618–880), was constructed based on the receptor-type protein tyrosine phosphatase delta (PDB ID: 4yh7.1.A). Sequence identity between the target and template was 28.69%. As shown in Figure 3(b), it predicted that the mutation affected the amino acid side chain by the substitution of valine acid to alanine.

### 3.3. Clinical Data

**Figure 4:**
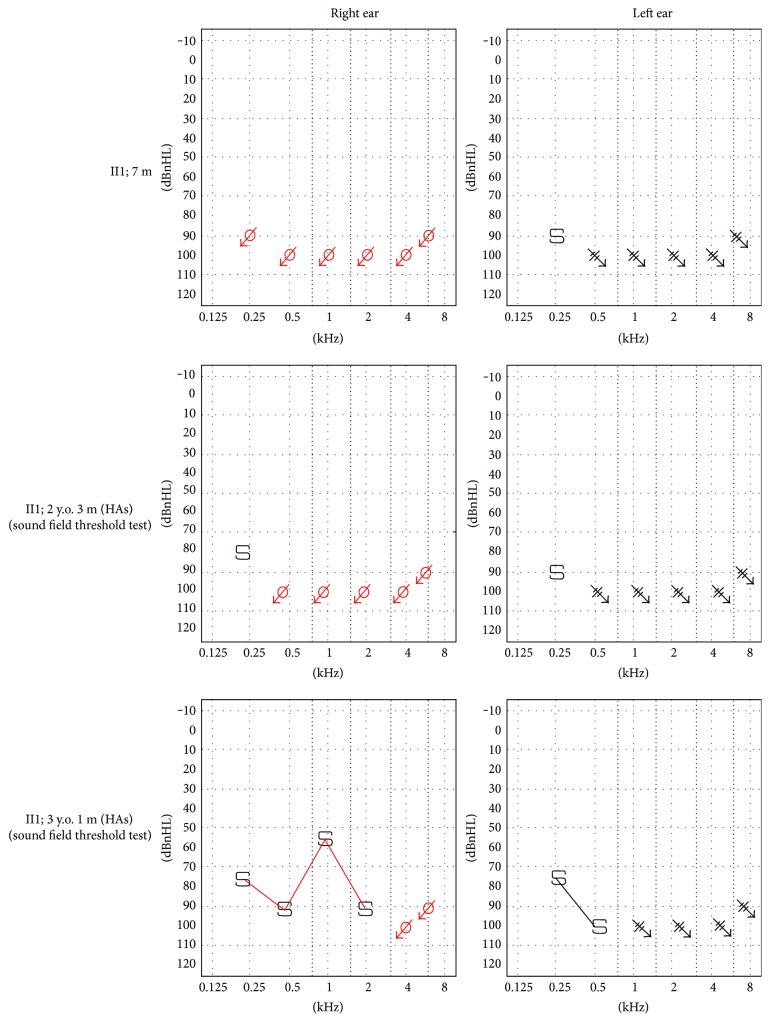
Pure tone audiometry. Visual reinforcement audiometry showed severe-profound SNHL at month 7. After hearing aids (HAs), sound field threshold test showed improvement of hearing levels.

Patient II1 is a 4-year and 2-month-old girl. Newborn hearing screening was failed at the age of 42 days. She had been referred to the Department of Otorhinolaryngology, Wuhan Union Hospital, for hearing examinations when 3 months. 40 Hz auditory steady-state evoked potential showed that the thresholds were 85 dBnHL at 500 Hz and 100 dBnHL at 1000 Hz of the left ear and 95 dB at 500 Hz and 95 dB at 1000 Hz of the right ear. Auditory brainstem audiometry (ABR) showed that reproducible wave cannot be elicited at 105 dBnHL bilaterally. The diagnosis of congenital severe-profound sensorineural hearing loss (SNHL) was made. Her parents took her for the hearing compensation in our hospital after four months. A visual reinforcement audiometry (VRA), computed tomography (CT) scan of temporal bone, and magnetic resonance (MR) imaging were performed. The VRA showed no response of both ears in all frequencies (Figure 4). The CT showed narrow of both internal auditory canal without any malformation of middle ear and osseous labyrinth. The MR showed that bilateral cochlear nerves cannot be identified well, considering a neurological maldevelopment, while there was no abnormality of the membranous labyrinth. She was consequently fitted for bilateral hearing aids. Her hearing was followed up in the following years (Figure 4). VRA revealed response of 90 dBnHL at 250 Hz of the left ear and 80 dBnHL at 250 Hz of the right ear at age 2 years and 3 months old. Acoustic immittance testing was within the normal limit. When she was 3 years and 1 month old, VRA showed response of 75 dBnHL at 250 Hz of the left ear and 60 dBnHL at 1000 Hz of the right ear. She had no balance manifestation, tinnitus, or vertigo.

## 4. Discussion

Hair cells are the essential sound receptors of auditory system. The hair bundle, comprised of about 100 actin-filled stereocilia, on the top of hair cells can help the sound transduction from mechanical signal to electrical signal. PTPRQ is a stereociliar membrane protein which belongs to the type III receptor-like protein tyrosine phosphatase (PTPase). It has three domains: the fibronectin type III- (FNIII-) like extracellular domain, the transmembrane domain, and the cytosolic PTPase domain, which have phosphatidylinositol phosphatase activity [11]. The PTPRQ mutant mice showed malformation of shaft connectors and immaturation of cochlear hair bundles which lead to deafness [12]. Here, we identified a novel compound heterozygous cosegregating mutation in the PTPRQ gene, c.4472C>T p.T1491M (maternal allele) and c.1973T>C p.V658A (paternal allele), as a probable cause of autosomal recessive congenital SNHL in a Chinese population by using an approach of next-generation sequencing and Sanger sequencing method. These two mutations have never been reported in previous studies.

There are 9 mutation variants of PTPRQ gene causing SNHL reported until now. They have been identified in different ethnic groups and countries, including c.1285C>T in Palestinian [7]; c.1491T>A in Dutch [2]; c1369A>G in Moroccan [2]; c3125A>G and c.5981A>G in Chinese [8]; c.166C>G, c4046T>C, and c.1261C>T in Japanese [9]; and c.16_17insT and c.2714delA in a Kazakh family of China [10]. Here, novel compound heterozygous mutations in PTPRQ, c.4472C>T and c.1973T>C, were identified by next-generation sequencing + Sanger sequencing method in patient II1. These two substitutions both occur within the extracellular FNIII domains, which can bind ligands like extracellular molecules and proteins [13, 14]. The mutations were predicted to perturb the amino acid side chain, which may interfere FNIII function and the interactions with other molecules and residues. Furthermore, based on the calculation results of SIFT and POLYPHEN2, the substitutions are deleterious and probably damage the protein functions. Although the c.1973T>C mutation rate is a little higher in normal population, which suggests that the pathogenicity is questionable, we cannot rule out the pathogenesis of the compound heterozygous mutations. Further experiments are needed to confirm our findings.

In this study, the patient II1 did not pass the examinations of newborn hearing screening when she was 42 days. In subsequent hearing tests, she went on to exhibit to a series of severe-profound sensorineural hearing loss symptoms. In the past studies, the affected patients had different hearing levels from moderate to profound and different progressions from stable to progressive. In our case, the patient had a severe-profound degree of hearing loss and a stable progression. Based on the results of existing PTPRQ mutation reports, we suspect that there is no obvious correlation between genotype and phenotype. PTPRQ mutation appeared to be liable for the vestibular dysfunction. The patients with PTPRQ mutations always had vertigo or dizziness. Consistent with this manifestation, the Ptprq^−/−^ mice showed defects in the hair bundles of the saccule and ampullae [15]. However, patient II1 in our study had no experience of vestibular dysfunction, which is the same as case 1 reported in Japan [9]. When patient II1 got to be 7 months, the CT and MR revealed a neurological maldevelopment of bilateral cochlear nerves. The relationship between the cochlear nerves abnormality and hearing loss is still not clear. These uncertainties need to be investigated further in animal models. In our case, the patient II1 received bilateral hearing aids at the age of 7 months. Her hearing threshold levels were advanced partly after hearing compensation using hearing aids. Consistent with our result, the hearing level of another patient with PTPRQ mutation was also improved after implantation at the age of 19 [9]. We speculated that hearing therapy might be helpful to the patients with PTPRQ mutations.

In summary, our findings suggest that the novel compound heterozygous PTPRQ mutations, c.4472C>T (p.T1491M) and c.1973T>C (p.V658A), are the cause of congenital SNHL in this family. The identification of additional mutations here further confirms the key role of PTPRQ in hearing function. More precise mechanism researches are needed for a better understanding of the gene.
